# Annotating Nontargeted LC-HRMS/MS Data with Two Complementary Tandem Mass Spectral Libraries

**DOI:** 10.3390/metabo9010003

**Published:** 2018-12-23

**Authors:** Herbert Oberacher, Vera Reinstadler, Marco Kreidl, Michael A. Stravs, Juliane Hollender, Emma L. Schymanski

**Affiliations:** 1 Institute of Legal Medicine and Core Facility Metabolomics, Medical University of Innsbruck, 6020 Innsbruck, Austria; vera.reinstadler@i-med.ac.at (V.R.); marco.kreidl@uibk.ac.at (M.K.); 2 Eawag, Swiss Federal Institute of Aquatic Science and Technology, 8600 Dübendorf, Switzerland; michael.stravs@eawag.ch (M.A.S.); juliane.hollender@eawag.ch (J.H.); 3 Institute of Biogeochemistry and Pollutant Dynamics, ETH Zurich, 8092 Zurich, Switzerland; 4 Luxembourg Centre for Systems Biomedicine (LCSB), University of Luxembourg, 4367 Belvaux, Luxembourg

**Keywords:** nontarget analysis, liquid chromatography mass spectrometry, compound identification, tandem mass spectral library, forensics, wastewater

## Abstract

Tandem mass spectral databases are indispensable for fast and reliable compound identification in nontargeted analysis with liquid chromatography–high resolution tandem mass spectrometry (LC-HRMS/MS), which is applied to a wide range of scientific fields. While many articles now review and compare spectral libraries, in this manuscript we investigate two high-quality and specialized collections from our respective institutes, recorded on different instruments (quadrupole time-of-flight or QqTOF vs. Orbitrap). The optimal range of collision energies for spectral comparison was evaluated using 233 overlapping compounds between the two libraries, revealing that spectra in the range of CE 20–50 eV on the QqTOF and 30–60 nominal collision energy units on the Orbitrap provided optimal matching results for these libraries. Applications to complex samples from the respective institutes revealed that the libraries, combined with a simple data mining approach to retrieve all spectra with precursor and fragment information, could confirm many validated target identifications and yield several new Level 2a (spectral match) identifications. While the results presented are not surprising in many ways, this article adds new results to the debate on the comparability of Orbitrap and QqTOF data and the application of spectral libraries to yield rapid and high-confidence tentative identifications in complex human and environmental samples.

## 1. Introduction

Tandem mass spectral databases are indispensable for fast and reliable compound identification in nontargeted analysis with liquid chromatography–high resolution tandem mass spectrometry (LC-HRMS/MS) [1,2,3,4,5,6,7]. These databases have been applied in diverse fields, including forensics, environmental analysis, food analysis, and metabolomics. They are usually applied for target and suspect analysis [8,9,10,11], and enable fast and automated annotation of components [12,13]. Database searching can yield identifications at a high confidence level. According to the scheme introduced by Schymanski et al. [14], a Level 2a identification (probable structure via spectral match) can immediately be reached with sufficient match to a library spectrum. Even Level 1 (structure confirmed by a reference compound) can be achieved when the library spectrum and associated retention time (or index) match with data acquired on the same analytical set-up as in the sample. This identification scheme was designed specifically for HRMS/MS data and is applied in the current manuscript. However, in the context of this article, these levels do not differ markedly from the Metabolomics Standard Initiative levels (MSI) 1 (Identified compounds) and 2 (Putatively identified compounds based upon spectral similarity with spectral libraries) [15]. 

Tandem mass spectral databases consist of two integral parts: (1) the collection of tandem mass spectral data accompanied by chemical information on the corresponding compounds, and (2) a database management system with diverse search functions. Tandem mass spectra are usually produced by collision-induced dissociation (CID) or higher-energy collision dissociation (HCD). The instruments most commonly applied for the acquisition of reference spectra are quadrupole time-of-flight (QqTOF) and iontrap/quadrupole-Orbitrap. Before storage, spectra are usually curated and cleaned employing multiple steps, which can include some or all of noise and artefact removal, peak annotation and recalibration, testing and benchmarking, as well as expert reviewing [16,17,18,19,20,21]. 

A challenge limiting tandem spectral database development has been the variability in observed fragmentation reactions caused by limited standardization and harmonization of experimental conditions. To cope with these reproducibility issues, state-of-the-art libraries contain multiple spectra per compound [17,22,23,24]. This is usually accomplished by comprehensive coverage of compound-specific breakdown curves via stepwise increase of applied collision energies. Combining these libraries with appropriate tailor-made search algorithms [25,26,27] enables reliable and robust identification. Such databases are characterized by false positive rates and false negative rates below 5% [3]. 

Tandem mass spectral libraries are constantly growing. The total number of compounds covered by tandem mass spectral databases is already in the range of several tens of thousands [1,2]. However, the overlap between libraries is still relatively limited [1]. While the results of extensive testing and benchmarking experiments will provide guidance for database selection [20], as has recently been investigated for genome-wide metabolic networks [28], such data is not available for the majority of established databases in an environmental context. A further complication is the fact that databases were established on either single or multiple instruments (i.e., QqTOF and various Orbitrap hybrid instruments). There are a range of scientific opinions on whether Orbitrap databases with HCD (and sometimes CID) spectra and QqTOF databases with CID spectra offer complementary identification possibilities. Initial findings suggest that HCD MS/MS spectra yield acceptable matches in CID mass spectral databases [29]. However, a thorough evaluation of the complementarity of these two important types of tandem mass spectral databases has not been accomplished yet.

Here, we use two specialized collections to investigate the complementarity of QqTOF and Orbitrap libraries, where the Orbitrap library contains both HCD and ion trap CID spectra. First, we investigate the comparability of the spectra in the two libraries, one created on a QqTOF in a forensic-toxicological context, the other a subset of Orbitrap spectra from MassBank compiled in an environmental context. We then use both libraries for mining nontarget Orbitrap and QqTOF data. While more extensive collections are available, we have limited this investigation deliberately to these specialized collections, as both the libraries and nontarget data were generated under relatively consistent conditions at the respective institutes of the coauthors, allowing greater intuitive interpretation of the results beyond other, more extensive collections where this institutional background knowledge is missing. 

## 2. Results and Discussion

### 2.1. Testing and Benchmarking of the Tandem Mass Spectral Libraries

In the first evaluation approach, the performance of the two well-established tandem mass spectral libraries was evaluated. The first collection was the “Wiley Registry of Tandem Mass Spectral Data”, hereafter termed WRTMD, developed on QqTOF instruments. The second library was the Eawag collection part of MassBank, developed on Orbitrap instruments (for more details see the “Materials and Methods” section). Overall, 14,693 QqTOF spectra representing 1349 compound species (i.e., including some compounds with multiple entries due to different precursor ions such as abundant isotopes, adducts, and in-source fragments) and 7415 Orbitrap spectra representing 744 compounds were available. For the QqTOF spectra, fragmentation was accomplished by CID at various collision energies. Out of the entire set of Orbitrap spectra, 321 spectra were acquired with CID at 35 NCE (nominal collision energy units), and 7094 spectra with HCD at various collision energies. 

The WRTMD collection of 14,693 CID QqTOF spectra of 1349 compounds has been investigated in multiple studies and the reliability of the search expressed as sensitivity and specificity has been demonstrated [3,16,20,24,25,27,29]. Although the database was tested with spectra acquired on all common types of tandem mass spectral instrumentation, the observed error rate was typically below 5%. This proven track record renders the WRTMD highly suitable for benchmarking experiments.

The Eawag collection of 7415 Orbitrap spectra (321 CID, 7094 HCD), representing 744 compounds, has a proven record of success in application work [8,9,10,30,31]. As described above, the library spectra are filtered and recalibrated [18]. This level of data curation also renders the Eawag collection suitable for quality tests. The influence of recalibration and cleanup of library spectra on database searching is shown in Table 2 of Stravs et al. [18]. 

Investigating the overlap of the two libraries tested revealed 233 compounds present in both collections. These 233 compounds represented 17.3% of the WRTMD (2840 QqTOF spectra) and 31.3% of the Eawag collection (2405 Orbitrap spectra). 

As described in the “Materials and Methods” section, the ‘MSforID Search’ was used for spectral matching to obtain *amp-* and *ramp*-values. The thresholds (see “Materials and Methods”) are deduced from quality tests and represent a compromise between sensitivity and specificity [20,25]. 

Firstly, the compatibility of the Eawag collection with the spectral matching via ‘MSforID Search’ was evaluated. The true positive rate obtained by matching each individual spectrum of the Eawag collection to the entire library was determined. Sensitivity (= true positive rate) was found to be 99.5%. The same test with the WRTMD yielded a sensitivity value of 99.7%. Secondly, each library was used as test set to characterize the reliability of a match to the other collection. For statistical evaluation, libraries were divided into positive and negative controls. By querying the WRTMD with the Orbitrap spectra, sensitivity was 88.0% (2405 test spectra) and specificity (= true negative rate) was 97.7% (5010 test spectra). Querying the QqTOF spectra against the Eawag collection gave a true positive rate of 91.5% (2840 test spectra) and a true negative rate of 98.0% (11,853 test spectra). The observed specificities correlated well to values observed with other test sets [3]. However, the observed sensitivities fell short of expectations. Based on previous results [29], matching Orbitrap spectra to WRTMD was expected to yield a true positive rate closer to 100%. 

**Figure 1:**
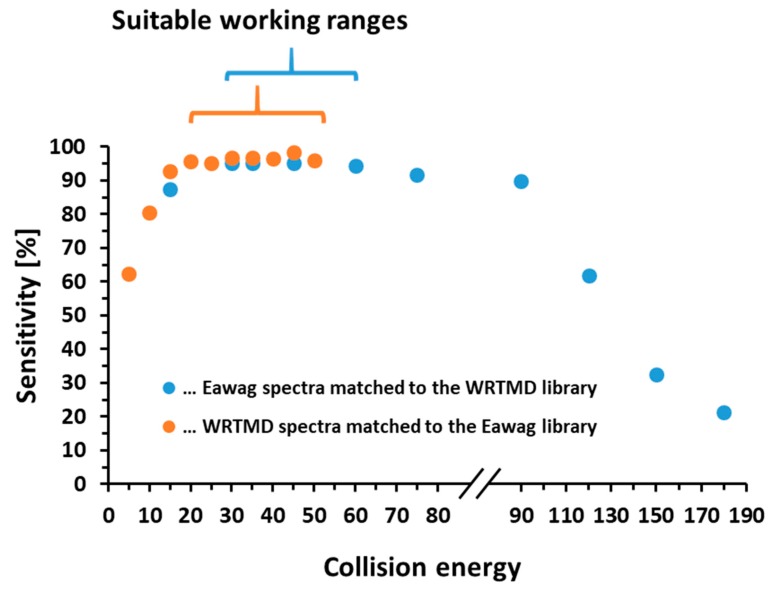
Evaluation of the reliability of a match in the WRTMD and the Eawag library illustrated by plots of sensitivity vs. collision energy applied during spectra acquisition.

As a result, the impact of the collision energy on the true positive rate was investigated to determine whether this could cause the reduced sensitivity (see Figure 1). For the majority of compounds, the collision energy ranges 20–50 eV on the QqTOF and 30–60 nominal collision energy units (NCE) on the Orbitrap seem to enable the acquisition of comparable reference and sample spectra. In these cases, substantial overlap between compound-specific spectra acquired on QqTOF and Orbitrap was observed. For the spectra acquired under these conditions, sensitivity values were 95.1–98.4%. For the QqTOF spectra acquired at very low collision energies (5 and 10 eV), sensitivity values fell below 81%. Similarly, for the Orbitrap spectra acquired at collision energies above 90, sensitivity values decreased to 21–61%. The ‘MSforID Search’ considers the similarity of the sample spectrum to the entire series of compound-specific reference spectra, such that the outcome is not a one-to-one match with a single reference spectrum. Thus, these results of this performance evaluation study indicate that to use these two libraries in a complementary manner in nontargeted LC-MS/MS identification, optimal sensitivity will be achieved for matching to both libraries if the nontarget data is acquired with collision energies in the range of 20–50 eV on a QqTOF or 30–60 NCE on an Orbitrap instrument, which was the case for the application cases presented below.

### 2.2. Compound Annotation Workflow for Application Samples

As discussed above, tandem mass spectral libraries are valuable for mining nontargeted LC-MS/MS data and can rapidly yield either Level 2a (library match) or Level 1 (in-house reference standard match) identifications. 

Workflows for mining nontargeted LC-MS/MS data usually involve diverse steps of feature detection, feature annotation, and compound identification. A feature detected by nontargeted LC-MS/MS is characterized by the *m/z* and retention time and, where available, the isotopic pattern of the precursor ion, any additional adduct species, and the corresponding fragmentation pattern. Particularly in environmental analysis and metabolomics, peak picking and extracted ion chromatograms (XICs) often play a key role in data processing. However, the data mining approach used for the plasma and wastewater sample here is different. All features containing information on the *m/z* of the precursor ion and the fragmentation pattern are matched directly to the tandem mass spectral library. This approach is suitable for complex data when searching using tandem mass spectral databases with high sensitivity. It also avoids the loss of matching compounds that may not have been detected by peak picking algorithms. 

### 2.3. Application Work

#### 2.3.1. Application 1: Systematic Toxicological Analysis of Human Plasma Samples 

Forensic toxicology is an important field of application for nontargeted LC-MS/MS [3,5]. Although the WRTMD has a proven record of success in forensic toxicological analysis [3], this library does not cover the full range of compounds principally observable in human samples and should therefore be complemented by other databases. To evaluate the impact of applying multiple libraries for compound annotation, 10 human plasma samples were submitted to systematic toxicological analysis involving nontargeted LC-MS/MS with data-dependant acquisition (DDA) on a QqTOF instrument. Tandem mass spectra were acquired at 35 eV with a collision energy spread of 10 eV. This CE is well within the working range defined above. The obtained data sets were then matched to the WRTMD and Eawag collections. False positive matches were sorted out by expert reviewing, which involved visual inspection of the spectral match. 

**Figure 2:**
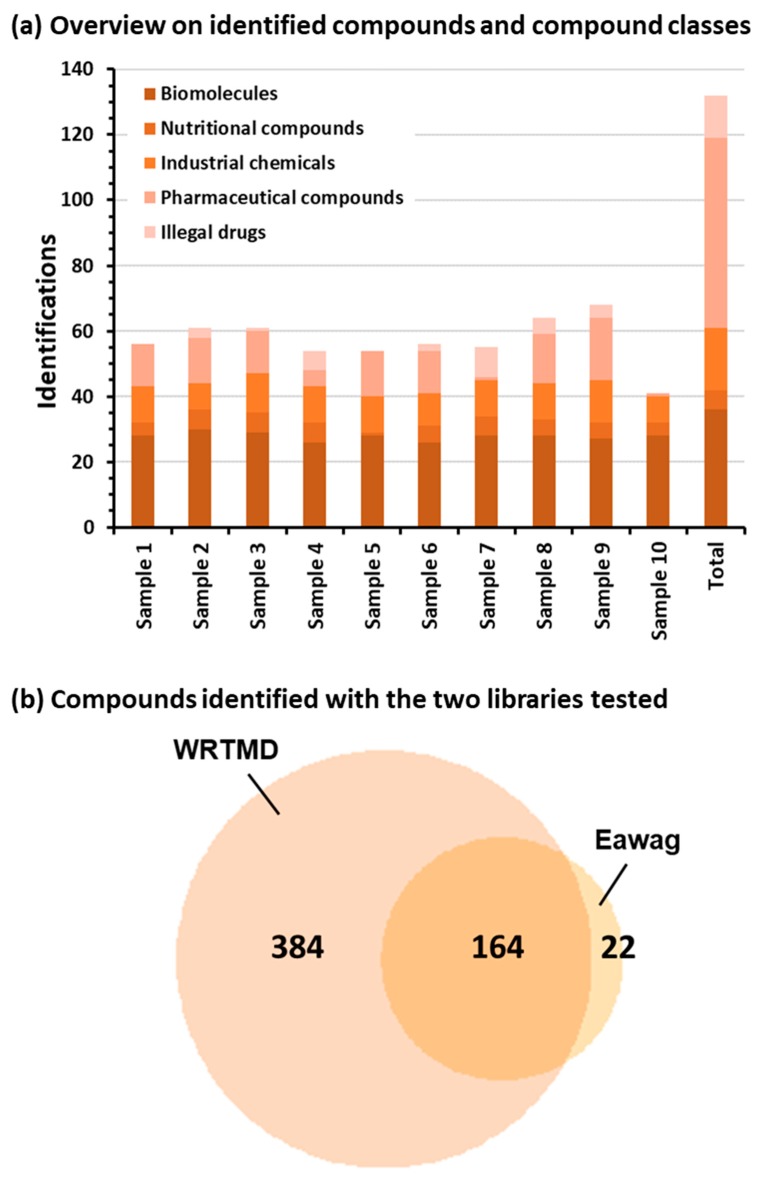
Application of the two tandem mass spectral libraries to systematic toxicological analysis of 10 authentic plasma samples. Nontargeted LC-MS/MS data was acquired on a QqTOF instrument using DDA. (**a**) Overview on the number of compounds identified in different compound classes via the combined use of the two libraries tested, and (**b**) the Venn diagram illustrating the number of identified compounds obtained with the two libraries tested.

In the 10 samples analyzed, a total number of 132 compounds were identified (Figure 2a, Supplementary Table S1). The number of identifications obtained for the individual samples ranged from 41 to 68. In each sample, a considerable number of endogenous compounds were detected. These biomolecules observed included amino acids, biogenic amines, steroids, nucleosides, and vitamins, which are only covered by WRTMD. Several nutritional compounds were observed, including caffeine, nicotine, and piperine, as well as their corresponding metabolites. A third group of observed compounds represented industrial chemicals. While some of these were also detected in the blank and thus may represent impurities and contaminants introduced after sample collection, there were nine compounds that were only observed in patient samples. These included the vulcanization accelerators 2-mercaptobenzothiazole and dibenzothiazyldisulfide, the corrosion inhibitor 2-hydroxybenzothiazole, the cosmetic ingredients ethylparabene, propylparabene, and octocrylene, the plasticiser benzyl butyl phthalate, as well as phenylurea and neocuproine. Detection of these industrial chemicals suggests that nontargeted LC-MS/MS techniques will be an important approach to detect unexpected compounds in human biomonitoring [32]. The fourth group of compounds detected were pharmaceutical compounds and corresponding metabolites. In total, 58 different species were detected. In accordance with previous findings [33], a high number of psychoactive drugs were observed, and these included 12 compounds belonging to the group of benzodiazepines and 8 to the group of opioids. Thirteen antidepressants and six antipsychotics were also identified. The last group of observed compounds represented illegal drugs and corresponding metabolites. Their detection provided evidence for cannabis consumption by four patients, cocaine consumption by six patients, and heroin consumption by one patient. There were three patient samples without any illegal drug detected. Further information about the identified compounds, including chemical identifiers, is given in the Supplementary Materials (Tables S1 and S4). 

An important aspect of this study was the evaluation of the number of compounds identified with the two libraries employed in the context of forensic toxicological analysis (Figure 2b). Out of the 570 identifications obtained, 384 (67.4%) were only obtained with the WRTMD, 22 (3.9%) only with the Eawag collection, and 164 (28.8%) with both libraries tested. Obviously for forensic samples, searching the Eawag collection enables verification of a considerable number of matches to the WRTMD, but it only provided a limited number of unique matches. This observation is quite reasonable taking into account that the Eawag library was initially built for environmental applications. The 164 identifications obtained with both libraries corresponded to 39 reference compounds. All other identifications involved compounds that were only included in one of the two libraries applied (85 compounds of the WRTMD and 9 compounds of the Eawag collection). 

#### 2.3.2. Application 2: Comprehensive Compound Identification in Wastewater Influent Samples Collected in a Local Wastewater Treatment Plant (WWTP)

Environmental analysis is another important field of application for nontargeted LC-MS/MS workflows [8,9,10,11,30,31]. Particularly in water analysis, the Eawag collection has a proven record of success. Recently, it has been demonstrated that the WRTMD is applicable for that purpose as well [34]. To evaluate the coverage of the two libraries, samples collected at the WWTP Rossau from 1–10 April 2016, were submitted for nontargeted LC-MS/MS analysis with DDA on a QqTOF instrument. Tandem mass spectra were acquired at 35 eV with a collision energy spread of 10 eV. This CE was well within the working range defined above. The obtained data sets were matched to the WRTMD and Eawag collections. False positive matches were sorted out by expert reviewing. 

**Figure 3:**
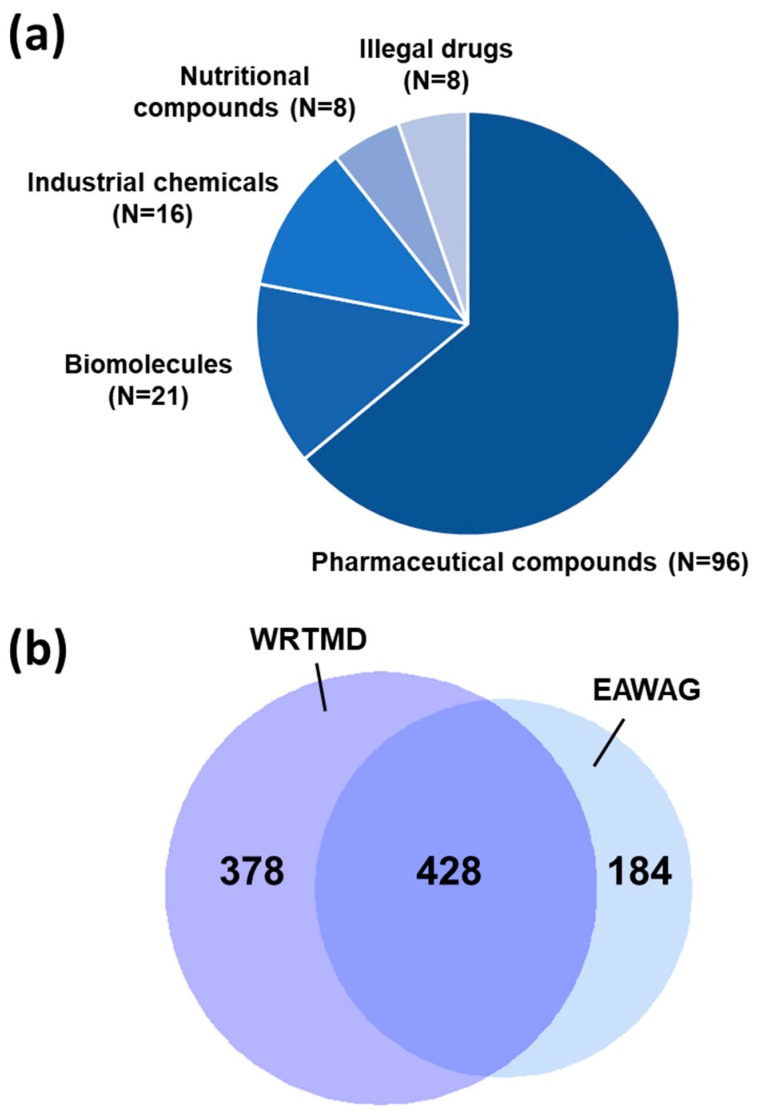
Application of the two tandem mass spectral libraries to the analysis of wastewater samples collected at the WWTP in Innsbruck. Ten influent samples were analyzed. The nontargeted LC-MS/MS data was acquired on a QqTOF instrument using DDA. (**a**) Overview on the number of compounds identified in different compound classes, as well as (**b**) a Venn diagram characterizing the number of identified compounds obtained with the two libraries tested are provided.

In the 10 influent samples, 149 different compounds were identified (Figure 3a, Supplementary Table S2). Pharmaceutical compounds and their metabolites represented the largest group of compounds detected (*N* = 96). Diverse antipsychotics, anticonvulsants, antidepressants, hypnotics and sedatives, hypoglycaemic agents, anti-inflammatory agents, cardiovascular agents, analgesics, and antibiotics were present. Clearly, wastewater analysis yields a comprehensive overview on medical prescription and consumption practices. Other important classes of compounds observed included biomolecules (*N* = 21) and industrial chemicals (*N* = 16). The groups of nutritional compounds (*N* = 8) and illegal drugs (*N* = 8) provide some insights into lifestyle of the community monitored. It provides evidence for the consumption of caffeine and tobacco, as well as of cocaine, amphetamine, MDMA, and heroine. 

A total of 990 identifications were obtained (Figure 3b) with the two libraries. The WRTMD produced 806 identifications, and the Eawag collection 612 identifications. Of these, 378 identifications (38.2%) were solely obtained by the WRTMD, 184 identifications (18.6%) solely by the Eawag collection, and 428 identifications (43.2%) by both libraries tested. This clearly proves that the two libraries complement each other in wastewater analysis. Thus, for more comprehensive compound identification (at Level 2a), the combined use of the two libraries is recommended. 

False negative rates were determined using the 449 identifications corresponding to compounds that were available in both libraries tested. The WRTMD produced 8 (1.8%), and the Eawag collection 13 false negative identifications (2.9%). In the majority of cases, the false negatives matched the corresponding reference compounds but were sorted out during data evaluation based on match probability values below the defined thresholds or during the final expert reviewing. Thus, when using stringent thresholds, the combined use of two or more libraries is recommended. The lower false negative rate for the WRTMD is most likely due to the fact that the acquisition data better matched the original library data. 

#### 2.3.3. Application 3: Retrospective Compound Identification in LC-MS/MS Data Acquired from Swiss Wastewater Effluent Samples 

The third set of experimental data was selected to evaluate the compatibility of data mining workflow presented here with Orbitrap data. The test sets were obtained from analysing nine Swiss wastewater effluent samples by nontargeted LC-MS/MS with DDA [30]. Tandem mass spectra were acquired at CID 35 and HCD 60. These CE values were within the working range defined above.

**Figure 4:**
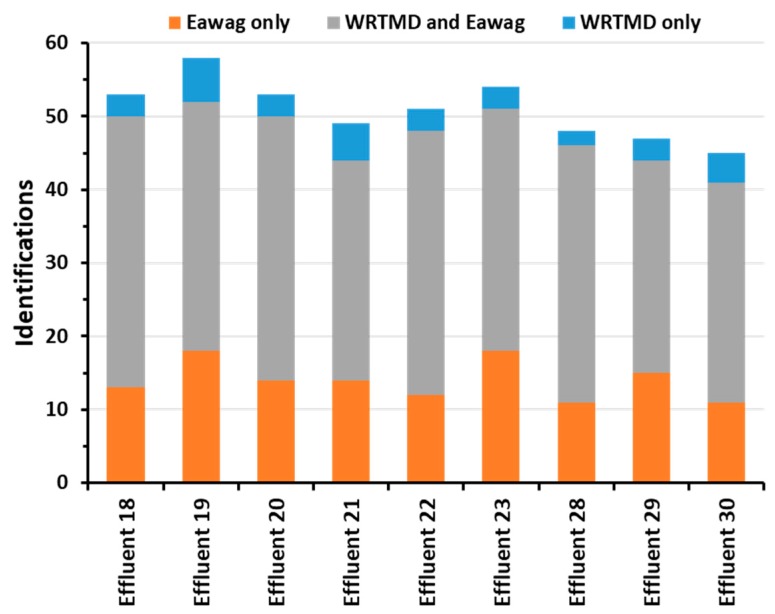
Application of the two tandem mass spectral libraries to the analysis of wastewater samples collected at the effluent of nine Swiss WWTP. The target and nontargeted LC-MS/MS data was acquired on an Orbitrap instrument using DDA. The column chart visualises the number of identifications obtained with the WRTMD and/or the Eawag library for each sample analyzed.

In the nine samples analyzed, 82 different compounds were identified (Supplementary Table S3). These included 54 pharmaceutical compounds, 24 industrial compounds, 3 nutritional compounds, and 1 illegal drug. Identifications per sample ranged from 45 to 58, leading to a total number of 458 identifications (Figure 4). Only 7.0% of the identifications were obtained with the WRTMD, 27.5% with the Eawag collection, and 65.5% with both collections. As each institution generally develops their reference standard collection (and thus libraries) for the local conditions and studies of interest, it is not surprising that the Eawag library results in more % identifications for the Swiss data set, and the WRTMD for the Austrian data sets. 

With the 492 identifications corresponding to compounds that were available in both libraries tested, false negative rates were determined. The WRTMD produced 20 (4.1%) and the Eawag collection 14 false negative identifications (2.8%). As above, in the majority of cases, negatively identified compounds matched to the corresponding reference compounds but were sorted out during data evaluation based on match probability values below the defined thresholds or during the final expert reviewing. The lower false negative rate for the Eawag collection in this case supports the conclusion above that fewer false negatives can be expected when the sample acquisition matches the library acquisition. Nonetheless, it is clear that libraries acquired on different instruments can provide valuable additional information in many cases. 

**Figure 5:**
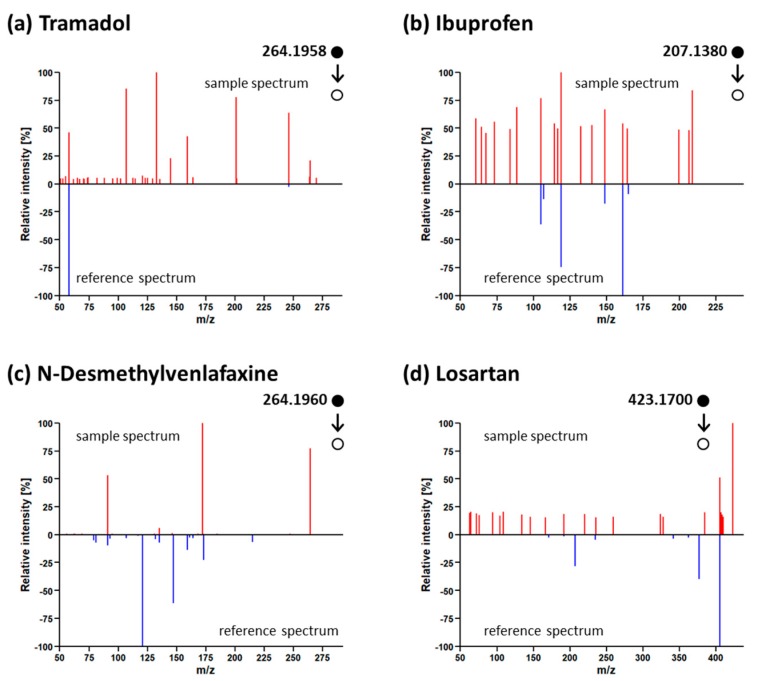
Examples of tandem mass spectra obtained from analysing wastewater samples with Orbitrap that showed insufficient spectral similarity to reference spectra of (**a**) tramadol (interfering peaks), (**b**) ibuprofen (noisy sample spectrum), (**c**) N-desmethylvenlafaxine (noise and/or interfering peaks), and (**d**) losartan (noisy spectrum and interfering peaks) stored in the Eawag collection. Black dots indicate precursor mass that triggered the MS/MS spectra (hollow dot).

As part of the initial study for this data set, a comprehensive quantitative target analysis was performed [30]. This analysis detected 73 compounds in positive mode. With the retrospective data analysis performed here, 58 of these targets were detected and identified. The identification of the remaining 15 targeted compounds was not reproduced. For six of these false negatives, the tandem mass spectral library search did not produce any evidence for their occurrence in the tested data sets (i.e., no fragmentation information was available). For the remaining eight compounds, at least one match was obtained, but in all cases, the spectral similarity was insufficient for a positive match (Figure 5). The observed discrepancies between the results obtained by target analysis and the suspect screening approach applied here can be explained by the different working principles of the two identification workflows. The suspect screening workflow relies on tandem mass spectral information for identification, such that compounds without fragments will not be detected—six compounds in this case, which were identified with retention time and exact mass and a correspondingly lower “identification point (IP) score” (2 IP vs 4.5 IP for targets with reported matching fragments) in the original study [30]. This means that some low-abundance but well-known compounds will be missed with a spectral library search approach. 

The most interesting cases are those that failed due to low spectral similarity values (see Figure 5). This is perhaps not surprising when querying spectra recorded in complex samples, as impurities are likely to occur even in MS/MS fragment information obtained using DDA [12]. This indicates some potential to apply a partial cleanup such as that performed in RMassBank prior to querying spectral libraries. A simple subformula or mass defect filter based on the precursor mass will potentially eliminate several interfering peaks that may correspond with different (coeluting) precursors that are still within the DDA window. Furthermore, this problem could be exacerbated with the increasing popularity of data-independent acquisition data (without precursor isolation and thus potentially more spectral interferences), increasing the need for deconvolution [12] and alternative data-processing approaches (e.g., [35]).

Another interesting result is that the retrospective data analysis performed in this study produced 67 additional tentative identifications corresponding to 24 compounds that were in WRTMD only and thus not obtained in the original investigation (with either target, suspect, or nontarget approaches) [30]. Two of these compounds (O-desmethyltramadol and tri(butoxyethyl)phosphate) were found to be among the thirty most abundant species observed in positive ion mode LC-MS/MS analysis.

Very recently, the Swiss wastewater data sets used here were included in a proof-of-concept study that demonstrated the potential of a global emerging contaminant early warning network to rapidly assess the spatial and temporal distribution of contaminants of emerging concern in environmental samples through performing retrospective data analysis [36]. The data sets were screened for 156 compounds included in the NORMAN Early Warning System (NormaNEWS) suspect list (http://comptox.epa.gov/dashboard/chemical_lists/normanews). With the data acquired in positive ion mode, 40 compounds were tentatively identified with the NormaNEWS method. For 31 of these compounds, reference spectra were available in the WRTMD and/or the Eawag collection and thus amenable to identification with the tandem mass spectral library search approach applied here. However, out of these 31 compounds detected in the wastewater samples, only 16 were successfully matched to the libraries with the approach used here. In the remaining cases, either no (*N* = 12) or only low-quality tandem mass spectra (*N* = 3) were available in the data sets (Figure 5), rendering confident compound identification (Level 2a or better) nearly impossible. This reinforces the need for high-quality spectral searching to provide additional evidence to increase the confidence of identification in nontarget screening efforts beyond the levels achieved with exact mass and retention time matches and, where available, selected fragment masses. As discussed above and as shown in Figure 5, the issue of interferences in the spectra extracted from complex samples played a role in the poor-quality spectra in many cases and a future investigation could look into whether spectral cleanup steps may improve these results.

## 3. Materials and Methods 

### 3.1. Tandem Mass Spectral Libraries

Two libraries were tested: the WRTMD (Wiley, Hoboken, NJ, USA) [37] and the Eawag collection in MassBank [18,23]. 

Tandem mass spectral data stored in the WRTMD were acquired on QqTOF instruments (Qstar XL or TripleTOF 5600+, Sciex, Framingham, MA, USA). For each reference compound, 10 or more product-ion spectra were acquired at different collision energy levels (resolution >10,000) to comprehensively cover compound-specific breakdown curves. Low-abundance and unspecific signals were removed from reference spectra by filtering [16,17]. For this study, a library version containing 1349 entries with 14,693 spectra was used. A more detailed description of the mass spectral library is provided on www.msforid.com. 

The Eawag library used for this study contained 7415 MS/MS spectra corresponding to 744 compounds. Reference spectra of 321 compounds were acquired on a LTQ-Orbitrap XL (Thermo Fisher Scientific, Waltham, MA, USA). For each of these compounds, HCD product-ion spectra were acquired at six different collision energy levels (HCD 15, 30, 45, 60, 75, 90) and a CID spectrum at one collision energy level (CID 35) to comprehensively cover compound-specific breakdown curves. The MS/MS spectra for each collision energy were recorded at two resolutions (7500 and 15,000). Reference spectra for a further 423 compounds were acquired on a QExactive Orbitrap (Thermo Fisher Scientific). For each of these reference compounds, HCD product-ion spectra were acquired at six different collision energy levels (HCD 15, 30, 45, 60, 75, 90). For a subset of 216 compounds, the collision energy range was extended to include HCD product-ion spectra at the collision energy levels 120, 150, and 180. MS/MS spectra were recorded at a resolution of 35,000. In all cases, the R package RMassBank was used to perform recalibration and cleanup of all spectra [18]. RMassBank can be downloaded from BioConductor, at http://bioconductor.org/packages/RMassBank/. The curated spectra (records published prior to 2018) are available at https://github.com/MassBank/MassBank-data/tree/master/Eawag. Listings of the chemicals available in MassBank.EU and WRTMD used in this investigation (beyond those detected and presented in the Supplementary Information) are given on the NORMAN Suspect Exchange (https://www.norman-network.com/?q=node/236) and CompTox Chemicals Dashboard (https://comptox.epa.gov/dashboard/chemical_lists). 

### 3.2. Tandem Mass Spectral Library Search

The library search was accomplished using the ‘MSforID Search’ [17,25]. The search algorithm determines the similarity between a sample spectrum and library spectra. The estimation of similarity starts with the identification of fragment ions that are present in both of the spectra being compared. These ions are called “matching fragments”. The spectral information retrieved is used to calculate the “reference spectrum specific match probability” (*mp*). As the mass spectral libraries contain multiple spectra per reference compound, multiple *mp* values per reference compound are obtained. To combine all these compound-specific *mp* values to one value that specifies the similarity between the unknown and the specific reference compound, the compound-specific *mp* values are averaged and normalized to yield the compound-specific “average match probability” (*amp*) and “relative average match probability” (*ramp*), respectively. These values range between 0 and 100. High compound-specific *amp* and *ramp* values indicate high similarity between the unknown and the reference compound. The substance with the highest *amp* and *ramp* value is considered to be the best match to the unknown compound.

Automated MSforID search was performed with a program written in Pascal using Delphi 6 for Windows (Borland Software Corporation, Scotts Valley, CA, USA; now Embarcadero Technologies, Inc., San Francisco, CA, USA) using the following search parameters: mass-to-charge ratio (*m/z*) tolerance of ±0.01, intensity cut-off factor of 0.01. The following criteria were used to classify obtained search results as tentatively correct positive results: precursor ion mass tolerance of ±0.01, *amp* > 1.0–10.0 and *ramp* > 30–50. The thresholds were determined using quality tests and represent a compromise between sensitivity and specificity [17,25]. The correctness of tentative identifications was checked by expert reviewing, which included visual inspection and comparison of tandem mass spectral data. 

### 3.3. Performance Evaluation

The performance of the two libraries (WRTMD, Eawag) was evaluated using two approaches. 

In the first approach, the libraries were searched against each other. Either library was used as reference or sample set. The spectra of compounds covered in both libraries served as positive controls. All other spectra represented negative controls. The positive controls were further grouped according to the collision energy settings used to acquire the individual spectra. For each test set, the number of positive identifications and the number of negative identifications were counted and used to calculate the statistical parameters sensitivity (= true positive rate) and specificity (= true negative rate). 

The second evaluation approach involved the analysis of forensic casework and environmental samples. Here, the focus was on evaluating the number and type of identifications obtained with the two libraries. The first set of samples analyzed represented 10 plasma samples collected as evidence in forensic casework at the Institute of Legal Medicine of the Medical University Innsbruck. The second set consisted of wastewater samples collected on 10 consecutive days (1–10 April 2016) at the WWTP Rossau (Innsbrucker Kommunalbetriebe AG, Innsbruck, Austria). The wastewater samples represented 24-h average samples of the influent [34]. The two sample sets were submitted to nontargeted LC-MS/MS on a QqTOF instrument (TripleTOF 5600+, Sciex, Framingham, MA, USA). Details of the analytical workflows employed are provided in the Electronic Supplementary File 1. The third set of samples consisted of nine Swiss WWTP effluent samples that had been analyzed by target and nontargeted LC-MS/MS on an Orbitrap instrument (LTQ Orbitrap XL, Thermo Fisher Scientific, Waltham, MA, USA). Details of the analytical workflow have been published previously [30]. Raw data files for the Swiss study are available at ftp://massive.ucsd.edu/MSV000079601. The remaining files cannot be uploaded for legal reasons, but can be made available to interested researchers upon request.

Data mining involved the extraction of the tandem mass spectra and a subsequent database search. Raw data files were converted to Mascot Generic Format (.mgf) files with the MSConvert from ProteoWizard [38]. The MS/MS spectra part of the .mgf files were extracted with a program written in ActivePerl 5.6.1 (Active State Corporation, Vancouver, Canada) to yield all MS/MS spectra as plain text (ASCII) files containing peak list information. These spectra were then submitted to the tandem mass spectral library search as described above. 

## 4. Conclusions

This article demonstrates the applicability of tandem mass spectral library searching to complex environmental and toxicological samples and reveals a wide range of comparability between collision energies of different tandem mass spectral instruments over a diverse range of compounds. For complementary use of the two libraries tested, the collision energy ranges 20–50 eV on the QqTOF and 30–60 NCE on the Orbitrap represented suitable working ranges. The results of the applications are in many ways unsurprising, that is, that searching in two libraries instead of one reveals more hits and that entries without fragmentation or with poor fragmentation information are not found. However, this article documents additional investigations to add to the debate on the comparability between QqTOF and Orbitrap instruments. This comparability is of utmost importance to achieve the desired goal of developing a unified and universally applicable tandem mass spectral database. Library development is laborious, time-consuming, and expensive, and this enormous effort is a serious hurdle for individual and isolated labs interested in contributing to accomplishing this mammoth task. Compatibility of libraries will enable the building of strong and dynamic consortia within scientific communities that will significantly increase the number of available reference spectra by sharing the connected workload. Further conclusions from this work are that the data mining approach used here could possibly be improved in the future through the application of some basic spectral cleanup to remove clear matrix interferences as well as the consideration of additional information such as isotope patterns/adduct and retention behavior.

## Supplementary Materials

The following are available online at http://www.mdpi.com/2218-1989/9/1/3/s1. Supplementary File 1: Experimental settings for nontargeted LC-MSMS analysis with QqTOF; Supplementary File 2: Excel spreadsheet containing the following tables: Supplementary Table S1: Overview of compounds identified by systematic toxicological analysis of ten authentic plasma samples; Supplementary Table S2: Overview of compounds identified by nontargeted LC-MS/MS in ten wastewater samples collected at the influent of the WWTP in Innsbruck; Supplementary Table S3: Overview of compounds identified by target and nontargeted LC-MS/MS in nine wastewater samples collected at the effluent of Swiss WWTPs; Supplementary Table S4: Additional chemical information for all compounds mentioned in Table S1 to S3.
