# Supplementary material for: Annotating Nontargeted LC-HRMS/MS Data with Two Complementary Tandem Mass Spectral Libraries
Source: Metabolites. 2018 Dec 23;9(1):3. doi: 10.3390/metabo9010003 (PMC6359582; doi:10.3390/metabo9010003)
Supplement: Supplementary file 1 [file metabolites-09-00003-s001.zip › Supplementary-File-1_Non-targeted_LC-MSMS_analysis_with_QqTOF_ES.pdf]

Supplementary File:

Annotating Non-targeted LC-HRMS/MS Data with Two Complementary Tandem Mass Spectral Libraries

Herbert Oberacher[1*], Vera Reinstadler[1], Marco Kreidl[1], Michael A. Stravs[2], Juliane Hollender[2,3], Emma L. Schymanski[2,4,*]

[1]Institute of Legal Medicine and Core Facility Metabolomics, Innsbruck Medical University, Innsbruck, Austria

[2]Eawag: Swiss Federal Institute of Aquatic Science and Technology, Dübendorf, Switzerland

[3]Institute of Biogeochemistry and Pollutant Dynamics (IBP), ETH Zurich, Zurich, Switzerland

[4]Luxembourg Centre for Systems Biomedicine (LCSB), University of Luxembourg, Belvaux, Luxembourg

***Corresponding authors:**

Assoc. Prof. Dr. Herbert Oberacher, Institute of Legal Medicine and Core Facility Metabolomics, Innsbruck Medical University, Muellerstrasse 44, 6020 Innsbruck, Austria. Tel.: +43 512 9003 70639, fax: +43 512 9003 73600, e-mail: herbert.oberacher@i-med.ac.at

Assoc. Prof. Dr. Emma Schymanski, Luxembourg Centre for Systems Biomedicine (LCSB), University of Luxembourg, Avenue du Swing 6, 4367 Belvaux, Luxembourg, T: +352 46 66 44 5616, E: emma.schymanski@uni.lu

Contents:

This file provides additional text to complement the Materials and Methods section in the main article. Additionally, an excel file is available with Tables S1-S4, detailing the results of the three case studies (Tables S1, S2 and S3) and chemical information for all compounds mentioned in the article (Table S4).

Overview on authentic samples analysed by non-targeted LC-MS/MS on a QqTOF instrument

Two sets of authentic samples were analysed to evaluate the performance of the two tandem mass spectral libraries (WRTMD, Eawag) tested within this study. The first set of authentic samples analysed represented ten plasma samples collected as evidence in forensic casework at the Institute of Legal Medicine of the Medical University Innsbruck. The second set consisted of wastewater samples collected on ten consecutive days (April 1st-10th 2016) at the wastewater treatment plant Rossau (Innsbrucker Kommunalbetriebe AG, Innsbruck, Austria). The wastewater samples represented twenty-four-hour average samples of the influent. The two sample sets were submitted to non-targeted LC-MS/MS on a QqTOF instrument (TripleTOF 5600+, Sciex, Framingham, MA, U.S.A).

Sample preparation workflows

Protein precipitation was used for processing of plasma samples. 125 µl plasma was mixed with 25 µl fencamfamine solution (60 ng/ml). Protein precipitation was accomplished by adding 375 µl acetonitrile. The mixture was vortexed for 20 s and put into an ultrasonic bath for 5 min. The liquid phase was separated by centrifugation (1900 g, 5 min). The liquid layer was transferred to a new vial and evaporated to

dryness at 25°C under a gentle stream of nitrogen. Finally, the dry residue was reconstituted in 25 µl 50% methanol (v/v) and submitted to LC-MS/MS analysis.

Wastewater samples were processed by solid phase extraction (SPE). 50 ml water was mixed with 50 µl fencamfamine solution (150 ng/ml). SPE was accomplished on Strata X 33 µm cartridges (200 mg/3 ml, Phenomenex, Torrance, USA). SPE columns were washed with 2 ml MeOH and equilibrated with 2 ml water. Next, the sample was rinsed through the column. After washing with 3 ml water and 2 ml 5% MeOH in water (v/v), columns were dried under vacuum for 15 min to enable elution with two times 750 µl of 2% FA in ACN (v/v). The eluate was evaporated to dryness at 25°C under a gentle stream of nitrogen. Finally, the dry residue was reconstituted in 50 µl aqueous 0.5% HOAc solution (v/v) and submitted to LC-MS/MS analysis.

Non-targeted LC-MS/MS analysis

For the analysis of plasma samples, separation of analytes was undertaken with a Waters ACQUITY UPLC system (Waters, Manchester, UK) using a Kinetex Biphenyl column (2.6 µm, 100 Å, 100 × 2.1 mm; Phenomenex) by applying a linear gradient of 2-98% acetonitrile in aqueous 0.5% acetic acid solution (v/v) within 15 min. The column temperature was held at 50 °C. The flow rate was set to 200 µl/min. 7.5 µl of sample were injected with partial loop overfill mode.

The chromatographic system for the analysis of wastewater samples was an Eksigent 425 LC system (Sciex, Framingham, MA, U.S.A). Separations were performed on a Luna C18 column (100 x 1.0 mm, 3 µm; Phenomenex, Aschaffenburg, Germany) by applying a linear gradient of 2-95% methanol in aqueous 0.05% acetic acid solution (v/v) within 10 min. The column temperature was held at 50 °C. The flow rate was set to 40 µl/min. The Eksigent Expert 400 autosampler (Sciex) was used for sample

injection. The injection volume was set to 5 µl.

For all samples, mass spectrometric detection was accomplished on TripleTOF 5600+ (Sciex). The mass spectrometer was operated in positive ESI mode using a DuoSpray ion source. The spray voltage was set to 5.5 kV. Gas flows of 40 arbitrary units for the nebulizer gas and 30 arbitrary units for the turbo gas were employed. The temperature of the turbo gas was adjusted to 200 °C. The instrument was operated at a mass resolution of ~30,000 for MS and ~15,000 for MS/MS, and automatically recalibrated every ten sample injections using APCI positive calibration solution delivered via a calibration delivery system (Sciex). The scan range was *m/z* 100-700 for MS, and m/z 50-700 for MS/MS. A duty cycle in the data-dependent acquisition mode included a single MS scan (accumulation time, 100 ms) followed by eight dependent MS/MS scans (accumulation time, 100 ms each) in the high sensitivity mode with dynamic background subtraction. The intensity threshold for triggering MS/MS experiments was set to 100 counts. MS/MS spectra were acquired at 35 eV with a collision energy spread of 10 eV. Former target ions were excluded for 30 seconds after two occurrences. The instrument was controlled by the Analyst TF 1.6 software (Sciex).
